# A Review on Medicinal Chemistry and Biological Activity of Dihydropyrimidinones Against HIV

**DOI:** 10.1002/ardp.70238

**Published:** 2026-04-10

**Authors:** Debora I. Leite, Fernando B. Macedo, Murilo Marinho de C. Lima, Isabelle S. Brum, Jonathan Fragoso Miranda de Oliveira, Ana Clara S. Costa, Stella C. Duarte, Sarah Moon, Stefany de Castro B. Moura, Andressa P. de Oliveira, Nubia Boechat, Monica M. Bastos

**Affiliations:** ^1^ Fundação Oswaldo Cruz, Instituto de Tecnologia em Fármacos, Farmanguinhos – Fiocruz, Laboratório de Síntese de Fármacos ‐ LASFAR Rio de Janeiro Rio de Janeiro Brazil; ^2^ Instituto Federal do Rio de Janeiro – IFRJ Rua Professor Carlos Wenceslau Rio de Janeiro Rio de Janeiro Brazil; ^3^ Programa de Pós‐graduação em Pesquisa Translacional de Fármacos e Medicamentos, Farmanguinhos (Fiocruz) Rio de Janeiro Rio de Janeiro Brazil; ^4^ Programa de Pós‐Graduação em Farmacologia e Química Medicinal (PPGFQM) Universidade Federal do Rio de Janeiro Rio de Janeiro Brazil

**Keywords:** AIDS, HIV, integrase, pyrimidinones, reverse transcriptase

## Abstract

Dihydropyrimidinones (DHPMs), also referred to as pyrimidinones, are privileged structures widely employed in the search for new compounds capable of inhibiting HIV replication. This core is found in natural alkaloids isolated from marine sponge species, which are known to inhibit the binding of the viral surface protein gp120 to the CD4 receptor of target cells. In addition, DHPMs exhibit a close structural relationship with nucleic acids, macromolecules that constitute the genetic material of both living organisms and viruses. Furthermore, the DHPM nucleus is present in several important anti‐HIV drugs that inhibit reverse transcriptase and integrase enzymes, indicating that the use of these privileged structures represents a faster and more promising approach for the development of novel HIV inhibitors. In this review, we provide an overview of DHPM‐based compounds as potential future trends in AIDS treatment, with a focus on studies published over the last 10 years. Overall, our analysis indicates that compounds containing the DHPM core generally display high potency against multiple HIV targets and may overcome antiviral resistance, reinforcing the relevance of this pharmacophoric fragment for the development of effective and innovative antiretroviral therapies.

## Graphical abstract

This article presents the latest advances in the development of dihydropyrimidinone (DHPM) derivatives as promising candidates against HIV. By exploring their interactions with essential viral targets and highlighting innovative compounds with potent antiviral activity, we provide an updated perspective on the opportunities for new antiretroviral therapies.

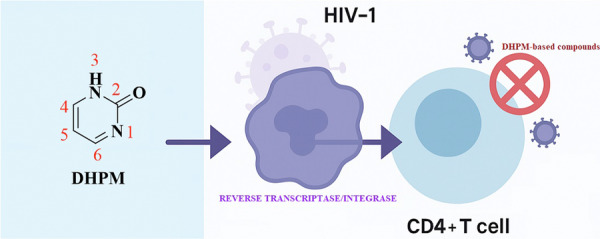

## 1 Introduction

**Figure 1:**
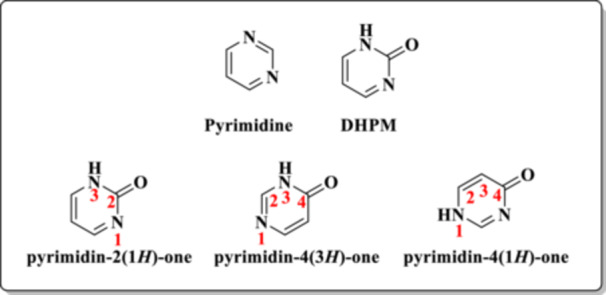
General structure of the pyrimidine rings and their derivatives, highlighting the most common positions of the carbonyl group in the DHPM core.

Dihydropyrimidinones (DHPMs), or pyrimidinones, differ from pyrimidines (Figure 1) by the presence of a carbonyl group in the ring, which can occupy three common positions within the heterocyclic ring (Figure 1). This structural similarity between DHPMs and pyrimidines confers certain shared chemical properties.

**Figure 2:**
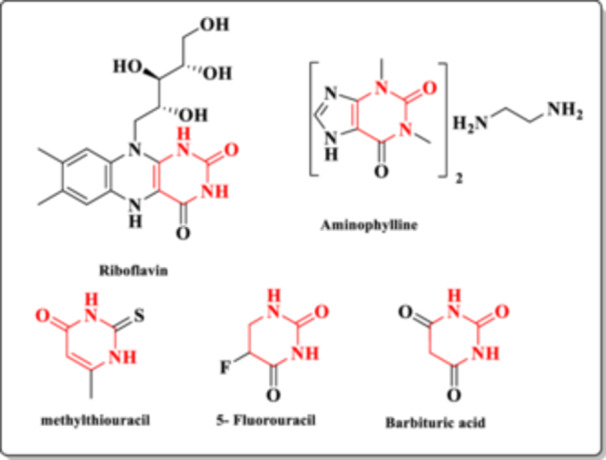
Drugs containing the DHPM core.

Many pharmacological profiles containing the DHPM core are described in the literature, including antihypertensive [1], antimicrobial [2], antibacterial, antifungal [3], anticancer [4], hypoglycemic [5], antiarrhythmic, anti‐inflammatory [6], analgesic [7], antituberculosis [8], DNA and RNA synthesis inhibitors [9], acetylcholinesterase inhibitors [10], and molecules targeting Alzheimer's disease [11]. This broad biological application results in the presence of the core in several drugs available on the market, such as riboflavin (vitamin B2), aminophylline (bronchodilator), 5‐fluorouracil (anticancer), methylthiouracil (antithyroid), and barbituric acid and derivatives (sedatives) (Figure 2) [12].

It is important to mention that the DHPM core is found in natural alkaloids isolated from sponge species, such as Batzeladine B, which is known to inhibit the binding of the surface protein gp120 of the human immunodeficiency virus (HIV) to the CD4 receptor of the target cell [13]. Thus, a prominent pharmacological profile of this class is its anti‐HIV activity [14].

**Figure 3:**
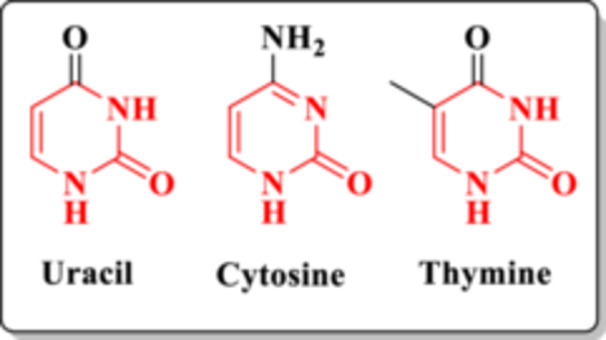
The DHPM core is present in the bases of DNA (cytosine and thymine) and RNA (uracil).

Moreover, due to their close structural relationship with nucleic acids (NAs) (Figure 3), DHPMs are considered privileged structures in the search for new compounds capable of inhibiting HIV replication. NAs are macromolecules of paramount biological importance, as they constitute the genetic material of both living organisms and viruses (DNA and RNA) and are involved in essential biological processes [15].

## 2 HIV and Its Relation to the DHPM Core

HIV is a retrovirus belonging to the *Lentivirus* genus that causes a progressive decline of the immune system, ultimately leading to acquired immunodeficiency syndrome (AIDS), which is characterized by gradual immune deterioration. More than 40 years after the first report of the disease, several drugs have been approved, and advances in antiretroviral therapy have substantially increased patient survival. Nevertheless, the rapid emergence of drug‐resistant viral strains remains a persistent challenge that must be continuously addressed [16].

In this context, the use of privileged structures with established antiretroviral activity represents a faster and more promising strategy for the discovery of new compounds active against HIV. Currently, the first‐line treatment recommended by the World Health Organization (WHO) and adopted in many countries consists of combination therapies targeting both HIV reverse transcriptase (RT) and integrase (IN) enzymes [17, 18]. Representative drugs from both therapeutic classes contain the DHPM core. Reverse transcriptase inhibitors (RTIs) are further classified into nucleoside/nucleotide RTIs (NRTIs/NtRTIs) and non‐nucleoside RTIs (NNRTIs).

**Figure 4:**
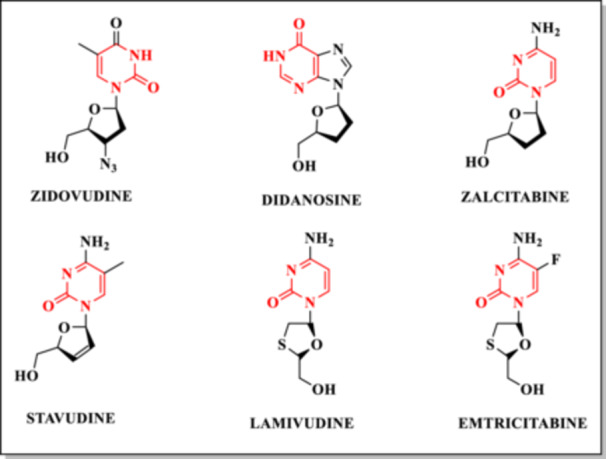
Chemical structure of some NRTIs containing the DHPM core (in red).

NRTIs/NtRTIs are analogs of endogenous nucleosides that, after intracellular triphosphorylation, compete with natural substrates for binding to the active site of RT. Their incorporation into the nascent viral DNA chain results in premature chain termination due to the absence of a hydroxyl group at the 3′ position of the sugar moiety. Representative examples of clinically approved NRTIs are shown in Figure 4 [19].

**Figure 5:**
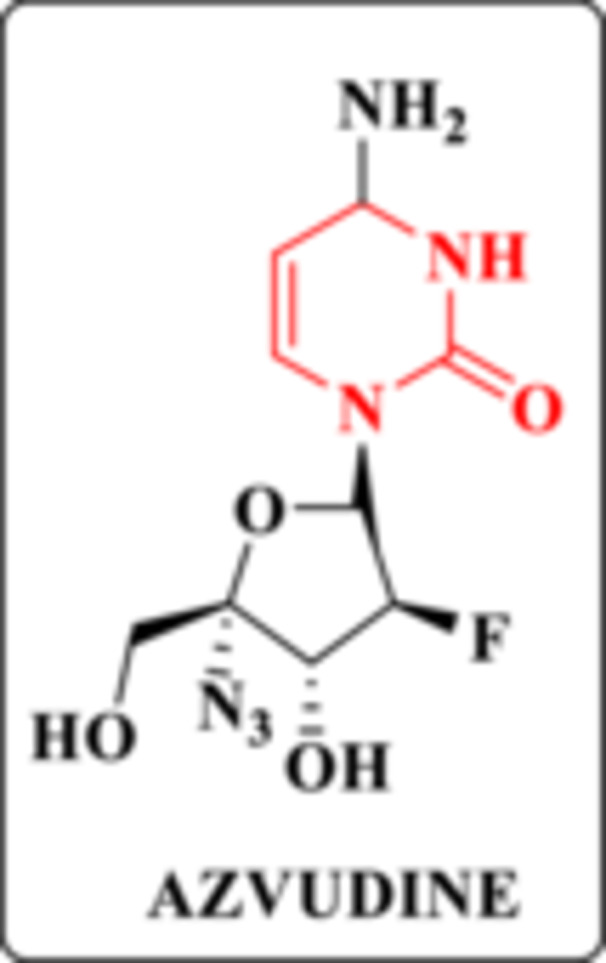
Chemical structure of FNC, highlighting the DHPM core.

Confirming the relevance of the NRTI/NtRTI class, in July 2021, the novel inhibitor azvudine (FNC) received conditional approval from the National Medical Products Administration of China for the treatment of HIV‐infected patients (Figure 5) [20]. FNC exhibits potent antiviral activity against both HIV subtypes, with effective concentration values for 50% of the maximal response (EC_50_) ranging from 0.03 to 6.92 µM for HIV‐1 and from 0.018 to 0.025 µM for HIV‐2. In addition, it retains activity against NRTI‐resistant strains [21]. A Phase II clinical trial evaluated its safety and efficacy; however, the results have not yet been disclosed [22].

**Figure 6:**
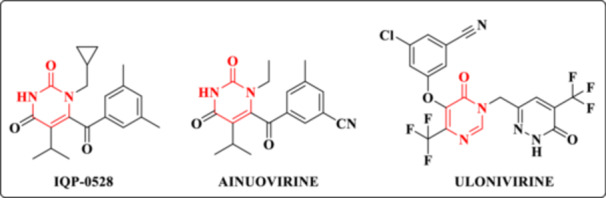
Chemical structure of new and potential NNRTIs containing DHPM.

NNRTIs exert their inhibitory activity by binding to an allosteric site within a hydrophobic pocket of RT, inducing conformational distortions that interfere with enzyme mobility and catalytic function. Several promising NNRTIs containing the DHPM core are currently under development, including IQP‐0528 and ulonivirine (Figure 6) [16, 21].

IQP‐0528 exhibits a dual mechanism of action, functioning both as an NNRTI and as an entry inhibitor against HIV‐1 and HIV‐2 [23]. This compound has been evaluated in humans as a 1% IQP‐0528 gel for pre‐exposure prophylaxis (PrEP). The results were favorable for its use as a topical microbicide, demonstrating local safety without systemic absorption, effective delivery of high local drug concentrations, and significant reductions in HIV infectivity in ex vivo assays. However, limitations include rapid clearance and limited penetration into vaginal tissues following rectal administration [24].

China has approved for clinical use an NNRTI featuring a DHPM core. Ainuvirine (Figure 6), which is currently under preclinical evaluation, has demonstrated potent in vitro antiviral activity against multiple HIV strains, as well as synergistic effects when combined with the NRTIs lamivudine (3TC) and tenofovir (TEN) [25, 26]. Moreover, ainuvirine exhibits a low potential for drug–drug interactions, favorable pharmacokinetic and pharmacodynamic profiles, good tolerability, and a low incidence of severe adverse effects. Notably, it reduces the neurotoxicity commonly associated with the first‐generation NNRTI efavirenz (EFV) and has shown superior efficacy compared with second‐generation NNRTIs, particularly under conditions of high viral load [27].

Ulonivirine is another NNRTI currently in Phase II clinical trials for HIV treatment, with the potential for once‐weekly administration. Preclinical studies have demonstrated that this derivative exhibits high antiviral potency [28].

To date, over 50 chemical scaffolds have been identified as potent NNRTIs, among which the HEPT (1‐[(2‐hydroxyethoxy)methyl]‐6‐(phenylthio)thymine) and DABO (dihydro‐alkoxy‐benzyl‐oxopyrimidine) classes are particularly noteworthy for incorporating the DHPM core [29].

**Figure 7:**
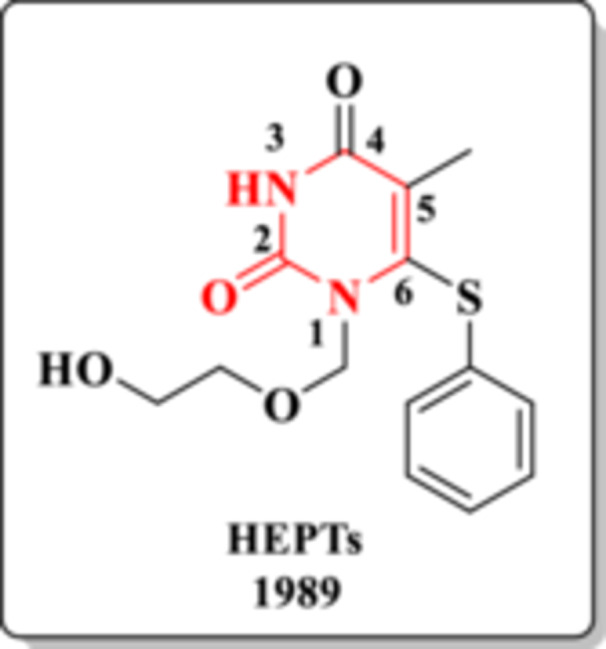
Chemical structure and year of discovery of HEPTs.

In 1989, Miyasaka et al. identified the HEPT scaffold (Figure 7) as the first candidate within the class of NNRTIs [30]. Biological evaluations demonstrated that this DHPM derivative inhibited HIV‐1 replication with an EC_50_ of 7 μmol/L, along with a favorable selectivity index (SI = 106) [31, 32].

**Figure 8:**
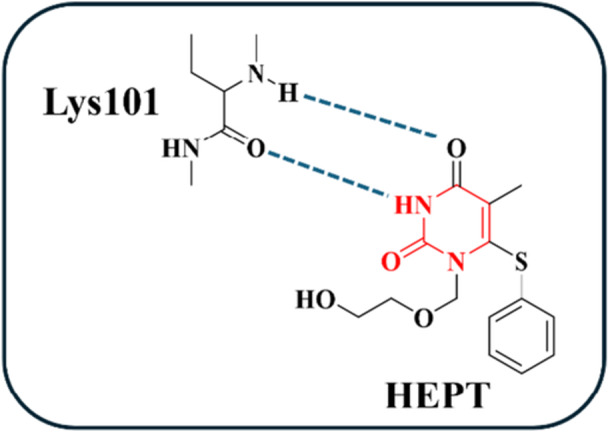
Interaction of HEPTs with the Lys‐101 amino acid residue through hydrogen bonds, represented by the blue dashed lines.

Compounds containing the HEPT core inhibit HIV‐RT by selectively binding to the enzyme's allosteric non‐nucleoside inhibitor binding pocket (NNIBP) [32, 33, 34]. This interaction disrupts the geometry of key polymerase residues, effectively locking the catalytic site of HIV‐RT in an inactive conformation and thereby impairing viral DNA synthesis [33, 35]. In HEPT derivatives, the DHPM core establishes hydrogen‐bond interactions—acting as both donor and acceptor—with the Lys101 residue located within the hydrophobic pocket of the RT allosteric site (Figure 8) [33].

It is noteworthy that most HEPT derivatives act selectively on HIV‐1 RT, showing little to no activity against HIV‐2. Historically, this class of compounds represents one of the most extensively studied among NNRTIs. The comprehensive body of research on HEPT‐based compounds has generated valuable insights for structure‐based optimization, guiding the development of increasingly potent HIV‐1 RT inhibitors [31].

Structure–activity relationship (SAR) studies have revealed that specific chemical modifications to the HEPT scaffold can confer dual inhibitory activity against both RT and IN. Consequently, considerable research efforts have been directed toward the design and development of potent dual inhibitors targeting these two essential HIV enzymes [32, 36, 37, 38, 39, 40, 41, 42, 43, 44, 45, 46].

**Figure 9:**
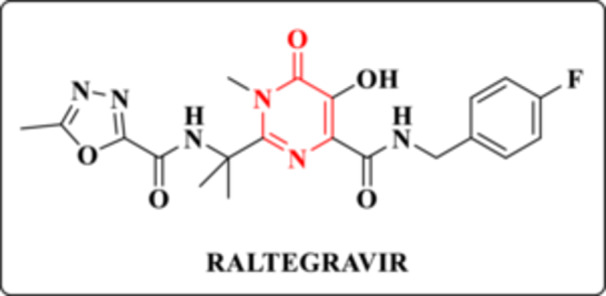
Chemical structure of the II, RAL.

HIV‐1 IN is the viral enzyme responsible for catalyzing the integration of proviral DNA into the host genome—an essential step in the viral replication cycle. Integrase inhibitors (IIs) exert their antiviral effect by blocking this process, and five such agents have been approved for clinical use to date. Among them is raltegravir (RAL), which notably contains a DHPM core (Figure 9) [21]. In 2015, Patel et al. provided a comprehensive review on the development of pyrimidinone‐based derivatives as IIs [47].

Inhibition of HIV‐1 IN is a process that relies on the presence of two Mg^2+^ ions, which act as essential cofactors within the enzyme's central catalytic domain (CCD). Interestingly, the catalytic site of the RNase H domain of HIV‐1 RT—responsible for hydrolyzing the RNA strand of the RNA–DNA hybrid—shares a similar structural topology with the CCD of IN. The hydrolytic mechanism involves water molecules acting as nucleophiles, assisted by magnesium ions coordinated with highly conserved residues in the RNase H active site (Asp443, Asp498, Asp549, and Glu478). Due to this structural and mechanistic similarity, IN and RNase H represent a promising dual‐target strategy for the development of multifunctional inhibitors [19]. In this context, recent studies have focused on the design and synthesis of novel DHPM derivatives as dual RNase H/IN inhibitors [36, 42, 44, 48].

**Figure 10:**
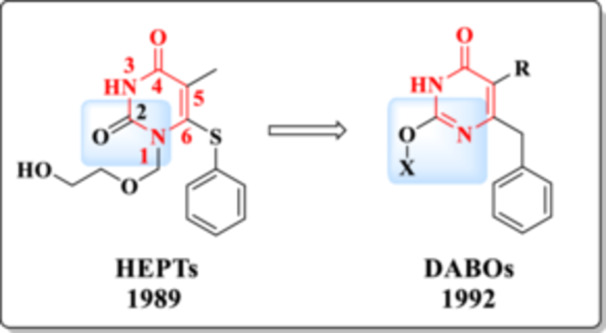
Structural similarity between HEPTs and DABOs. The modified parts are highlighted in blue.

The second major class of DHPM‐based compounds, extensively described as HIV‐1 NNRTIs, is the DABOs. DABOs exhibit notable structural similarity to HEPT derivatives, particularly due to the presence of the privileged pyrimidinone scaffold. The key structural distinction lies in the relocation of the substituent from the *N*‐1 position to the C‐2 carbon (Figure 10), a modification that results in significantly enhanced anti‐HIV activity [49, 50].

DABO derivatives play a pivotal role in the search for novel HIV‐1 inhibitors, as they have been reported to exhibit potent activity against both wild‐type and drug‐resistant HIV‐1 strains, as well as notable activity against HIV‐2 [51]. Crystallographic studies have demonstrated that DABO compounds can adopt multiple conformations within the RT binding pocket. This conformational flexibility enables the repositioning and reorientation of key functional groups, which is critical for preserving inhibitory potency across a broad spectrum of HIV variants, including those resistant to currently approved NNRTIs [51].

Since the discovery of DABOs in 1992 [52], numerous structural optimizations have been carried out on the DHPM core to develop novel and more potent NNRTIs with improved resistance profiles and enhanced pharmacokinetic properties. These modifications aim to fine‐tune the molecular interactions within the RT binding pocket, thereby increasing antiviral efficacy and minimizing the emergence of resistant HIV strains [31, 32, 50, 53, 54, 55, 56, 57, 58, 59, 60, 61, 62, 63]. Building on these structures, Nawrozkij et al. conducted a detailed SAR analysis of the DABO series to guide the rational design and optimization of novel HIV inhibitors with improved potency and resistance profiles [60].

The broad bioactive potential of DHPMs, widely recognized in the field of drug discovery, has driven the development of numerous synthetic methodologies for their preparation [15, 64, 65]. Among these, particular attention has been given to multicomponent reactions (MCRs), especially the Biginelli reaction, which was first described in 1893 by Pietro Biginelli [66].

This methodology enables the straightforward generation of a wide range of DHPM‐containing compounds via MCRs, provided that appropriate reagents are employed. In 2022, Sánchez‐Sancho et al. reviewed alternative synthetic routes for accessing DHPM derivatives, termed *Biginelli‐like* reactions, and provided an update on recent methodological advancements in this area [67]. Furthermore, the DHPM core has been recognized as a highly promising scaffold for the development of novel antiretroviral agents active against HIV‐1 [50].

In this review, we have summarized the key applications of DHPMs as a privileged scaffold in the development of potential anti‐HIV drug candidates, particularly targeting HIV‐1 RT and IN. We provide an overview of DHPM‐based compounds as emerging trends in AIDS therapy, with an emphasis on research conducted over the past decade.

## 3 Anti‐HIV Biological Activities of DHPM Compounds

**Figure 11:**
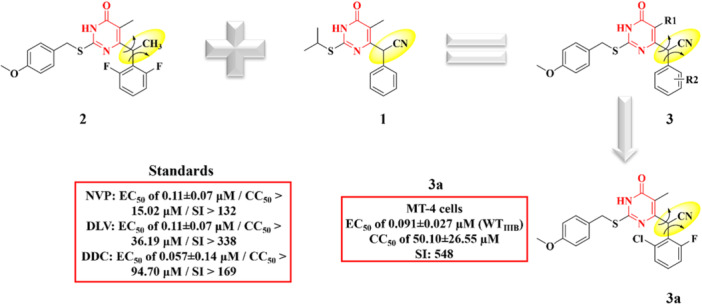
Design and anti‐HIV‐1 profile of Hybrid **3a**.

A class of DHPM‐related compounds have gained prominence as potent inhibitors of HIV‐1 RT. In a previous study by Wu and colleagues [68], a new series of DHPM was developed, among which Compound **1** (Figure 11) emerged as the most potent, exhibiting an EC_50_ value of 2 nM. Molecular docking studies revealed that the α‐cyanobenzyl group of this compound fits snugly within the aromatic‐rich NNIBP, engaging in favorable interactions with key residues including Tyr181, Tyr188, Phe227, and Trp229, as well as hydrophobic contacts with Leu234 and Pro95 (Figure 11).

Considering that the presence of an α‐cyano (α‐CN) group at the benzylic position could enhance π–π stacking interactions with the surrounding aromatic residues—while also promoting a conformational restriction similar to that observed in Compound **2**—the authors strategically combined the structural features of Compounds **1** and **2** to design a new series of hybrid molecules (Compound **3**, Figure 11). Compound **3a** emerged as the most promising candidate in the series, demonstrating an EC_50_ value of 0.091 ± 0.027 μM against wild‐type HIV‐1 (HIV_WTIIIB_) in MT‐4 cells. Its potency surpassed that of the reference drug zalcitabine (DDC), which exhibited an EC_50_ of 0.057 ± 0.14 μM, and was comparable to other standard NNRTIs, namely nevirapine (NVP) and delavirdine (DLV), both with EC_50_ values of 0.11 ± 0.07 μM. Importantly, this novel DHPM compound did not display significant cytotoxicity relative to the standards evaluated (Figure 11).

Furthermore, HIV‐1 RT inhibition assays demonstrated that the conformationally restricted hybrids exhibited potent inhibitory activity against the enzyme, with overall efficacy surpassing that of NVP, which showed an IC_50_ of 1.78 μM. Notably, Compound **3a** stood out as the most active inhibitor in the series, presenting an IC_50_ value of 0.17 μM.

Compound **3a** exhibited an excellent SI (548) (Figure 11), surpassing most of the clinically used reference drugs evaluated under the same experimental conditions. This SI value is markedly higher than that of DDC (SI = 169), NVP (SI = 132), and DLV (SI = 338), indicating that **3a** combines potent antiviral activity (EC_50_ = 0.091 μM) with remarkably low cytotoxicity toward MT‐4 cells (CC_50_ > 50 μM). This broad therapeutic window highlights a key advantage of **3a** and reflects its highly favorable balance between efficacy and cellular tolerability. The enhanced selectivity also suggests more specific interactions with the HIV‐1 RT binding pocket, consistent with the docking data showing stabilizing hydrogen bonds with Lys101 and strong π–π contacts with aromatic residues such as Tyr181 and Tyr188. Overall, the SI profile of **3a** reinforces its promise as a lead candidate within the conformationally restricted *S*‐DABO series and supports further optimization within this chemical scaffold.

Khalifa and Al‐Omar reported the synthesis and biological evaluation of a series of DHPM‐based compounds, designed as NNRTIs targeting HIV‐1 replication [69]. To develop this new class of compounds, the authors introduced various substituents at the C‐2 position of the DHPM core. Antiviral activity and cytotoxicity were assessed in MT‐4 cell cultures, employing MCK‐442 (IC_50_ = 0.005 μM; CC_50_ = 141 μM) and zidovudine (AZT) (IC_50_ = 0.04 μM; CC_50_ = 52 μM) as reference controls. The synthesized derivatives exhibited notable inhibitory activity against HIV‐1, with IC_50_ values ranging from 35.60 to 0.32 μM.

**Figure 12:**
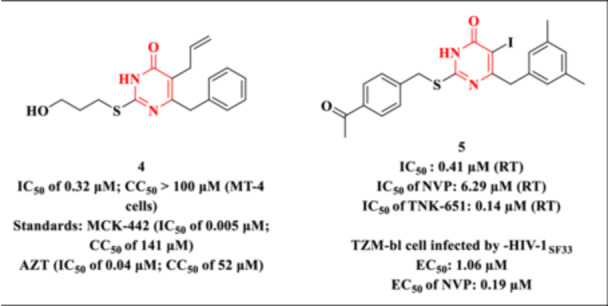
The anti‐HIV profile of the Molecules **4** and **5**.

Among the evaluated compounds, Molecule **4** (Figure 12) exhibited the highest antiviral potency (EC_50_ = 0.32 μM), together with a favorable cytotoxicity profile (CC_50_ > 100 μM), resulting in an SI greater than 312. Although this value suggests a promising therapeutic window, particularly when compared with reference drugs such as AZT (SI = 1300) and MKC‐442 (SI = 28,000), the authors do not provide a detailed analysis or discussion of the biological and pharmacological relevance of the SI. The absence of such discussion limits a critical interpretation of the compound's therapeutic potential, as the SI represents a key parameter in balancing antiviral efficacy and cellular toxicity.

Zhang et al. reported novel DHPM analogs designed to improve anti‐HIV‐1 activity [70]. The authors hypothesized that incorporation of an oxygen atom into the C‐2 side chain of the DHPM nucleus could enable additional hydrogen‐bonding interactions, potentially enhancing binding affinity. Moreover, extension of the π–π conjugated system through the introduction of electron‐withdrawing groups at the *para* position of the phenyl ring was proposed as a strategy to improve antiviral efficacy [71]. Optimization of the phenylmethyl moiety at the C‐6 *meta* position was also explored to promote closer interactions within the NNBP. Based on previous findings from the same group [72, 73], the presence of a halogen atom at the C‐5 position was postulated to facilitate interactions with the carbonyl group of Tyr181, thereby contributing to enhanced inhibitory activity against HIV‐1 RT.

To explore the SAR of this class, a series of 2‐arylalkylthio‐5‐iodo‐6‐substituted‐benzyl‐pyrimidin‐4(3*H*)‐ones (Figure 12) was synthesized and biologically evaluated. The compounds were tested for RT inhibition and in vitro antiviral activity using TZM‐bl cells infected with HIV‐1_SF33. Among the series, Compound **5** (Figure 12) exhibited the highest RT inhibitory activity, surpassing NVP and approaching the potency of the reference compound TNK‐651. However, in cell‐based assays, their efficacy was lower than that of the reference standard, indicating potential limitations in cellular uptake or metabolic stability. However, the authors do not report or discuss CC_50_ values or the corresponding SI. This lack of information hampers a comprehensive assessment of the compound's therapeutic window.

The SAR was further elucidated by the authors through molecular dynamics (MD) simulations, employing the crystallographic structure of HIV‐1 RT complexed with TNK‐651 (PDB ID: 1RT2). The computational studies revealed that the target compounds could form a key hydrogen bond between the N3‐H group of the pyrimidinone ring and the Lys101 residue of RT, a critical interaction for antiviral activity. Additionally, the presence of an iodine atom at the C‐5 position enabled the formation of a halogen bond with the carbonyl group of Tyr181, which significantly enhanced binding affinity and, consequently, antiviral potency. Molecular docking suggests that electronegative substituents at the C‐5 or C‐6 positions of the pyrimidine ring modulate the electronic density of the system, favoring proper ligand alignment and strengthening these polar interactions.

Interestingly, the incorporation of an oxygen atom in the C‐2 side chain introduced a further hydrogen bonding interaction with Tyr318 of RT. This additional hydrogen bond was proposed to serve as a pivot point, potentially allowing the compound to adopt a favorable conformation within the NNBP, thereby optimizing its interaction network with the enzyme [70].

**Figure 13:**
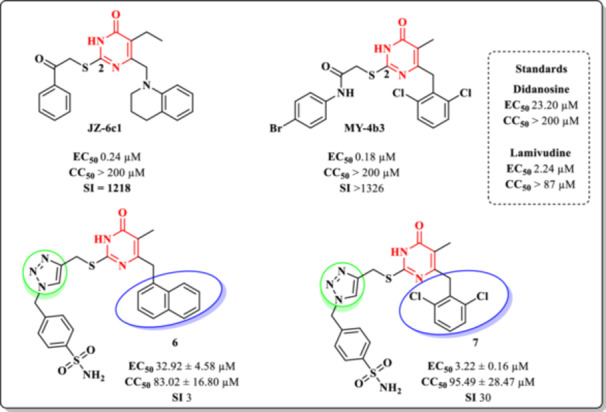
Anti‐HIV‐1 activity (EC_50_, against HIV‐1_IIIB strain) and cytotoxicity (CC_50_, in MT‐4 cells) of selected DHPM compounds: JZ‐6c1, MY‐4b3, Compound **6**, and Compound **7**.

In 2015, Fang et al. synthesized a series of DHPM molecules bearing a 1,2,3‐triazole substituent at the C‐2 position and evaluated their anti‐HIV activity in vitro [74]. The same research group had previously identified analogs with promising antiviral activity, including compounds MY‐4b3 (EC_50_ = 0.18 µM) and JZ‐6c1 (EC_50_ = 0.24 µM) (Figure 13) [75].

Based on SAR studies reported by Radi et al. [76], which highlighted the impact of C‐2 substitutions on antiviral efficacy, the authors proposed the introduction of a 1,2,3‐triazole ring as a bioisosteric replacement for the amide group. This modification was expected to enhance ligand–receptor interactions through stronger dipole–dipole and hydrogen bonding contributions. As a result, two distinct series of compounds were developed: one featuring a naphthalene moiety at the C‐6 position of the DHPM core (Series 6), and the other incorporating a 2,6‐dichlorobenzyl group at the same position (Series 7) (Figure 13).

The antiviral activity of the synthesized compounds was assessed in MT‐4 cells infected with HIV‐1_IIIB_. Regarding SAR observations, substitution at C‐6 with the 2,6‐dichlorobenzyl group (Series 7) proved beneficial, whereas the presence of a naphthalene moiety (Series 6) was detrimental to activity. In Series 6, only Compound **6** displayed measurable antiviral activity (EC_50_ = 32.92 ± 4.58 μM; CC_50_ = 83.02 ± 16.80 μM). In contrast, Series 7 yielded several compounds with micromolar activity, among which Compound **7** was the most potent, exhibiting an EC_50_ of 3.22 μM and a CC_50_ of 95.49 μM (Figure 13).

Although compounds from Series 7 exhibited micromolar antiviral activity and moderate cytotoxicity profiles, their SI remain significantly lower than those reported for the reference Compounds **JZ‐6c1** (SI = 1218) and **MY‐4b3** (SI > 1326) (Figure 13). These reference standards display a substantially wider therapeutic window, reflecting a more favorable balance between antiviral potency and low cellular toxicity. Therefore, despite the improvements observed for Series 7 compared to Series 6, the obtained SI values indicate that further structural optimization is required to achieve selectivity levels comparable to those of the reference compounds.

The search for more effective molecules led Li et al. to investigate the impact of various substitutions at the C‐2, C‐5, and C‐6 positions of the DHPM core [77]. The introduction of an adamantylmethyl group at C‐6, recognized as a pharmacologically relevant moiety due to its antiviral potential, was combined with modifications at C‐2, particularly the arylcarbonylmethylthio group, previously reported to enhance anti‐HIV activity—and small alkyl groups (methyl or ethyl) at C‐5. The antiviral potential of the resulting compounds was evaluated against wild‐type HIV‐1 (IIIB), a double mutant strain (RES056, K103N/Y181C), and HIV‐2 (ROD strain) in MT‐4 cells.

**Figure 14:**
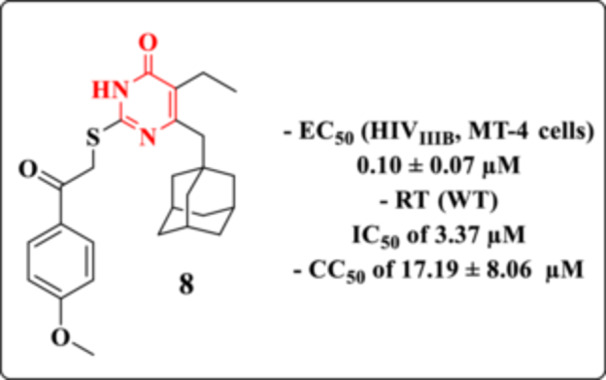
Anti‐HIV‐1 profile of Derivative **8**.

Most of the compounds exhibited moderate activity against the HIV‐1_IIIB_ strain, with EC₅₀ values ranging from 0.10 to 5.39 µM. Among them, Compound **8** (Figure 14) emerged as the most potent, with an EC_50_ of 0.10 µM, surpassing the activity of the reference drug NVP (EC_50_ = 0.26 µM), though remaining less effective than other clinically used NNRTIs such as DLV, EFV, and etravirine (ETR). However, none of the synthesized derivatives demonstrated inhibitory activity against the RES056 mutant strain or HIV‐2. With respect to the SI, the compound exhibited an SI value of 172. In contrast, the reference compounds evaluated under the same conditions showed markedly higher SIs, including > 58 for NVP, > 1054 for AZT, 950 for DLV, > 1218 for EFV, and 724 for ETR. These comparisons highlight that, despite the observed antiviral activity, the selectivity of the evaluated compound remains inferior to that of clinically established antiretroviral agents, underscoring the need for further optimization to improve its therapeutic window.

SAR analysis revealed that the ethyl substituent at C‐5 conferred superior antiviral potency compared to the methyl group, likely due to enhanced hydrophobic interactions within the RT binding pocket. Additionally, the arylcarbonylmethylthio moiety at C‐2 significantly contributed to antiviral activity, whereas the phenylaminocarbonylmethylthio analogs exhibited slightly diminished efficacy. Among para‐substituted derivatives at the phenyl ring, the sulfonamide group proved to be the most effective in inhibiting HIV‐1 replication.

Compound **8** demonstrated potent inhibition of the RT enzyme (IC_50_ = 3.37 µM) (Figure 14), outperforming both NVP (IC_50_ = 8.78 µM) and ETR (IC_50_ = 3.10 µM), confirming its high affinity as an NNRTI. Molecular docking simulations based on the HIV‐1 RT crystal structure (PDB code: 3DLE) provided further insights into the binding mode. The N‐3‐H group of the DHPM core forms a key hydrogen bond with the carbonyl group of Lys101, which is essential for RT binding affinity and inhibition. The ethyl group at C‐5 is situated in a hydrophobic pocket defined by the side chains of Tyr181 and Tyr188, while the C‐2 substituent is oriented into a spacious cavity bordered by Val106, Pro236, and Trp227. Notably, the adamantylmethyl moiety at C‐6 fits well within a hydrophobic region composed of residues Tyr181, Tyr188, Phe227, and Trp229, establishing favorable van der Waals and hydrophobic interactions with the RT enzyme [77].

**Figure 15:**
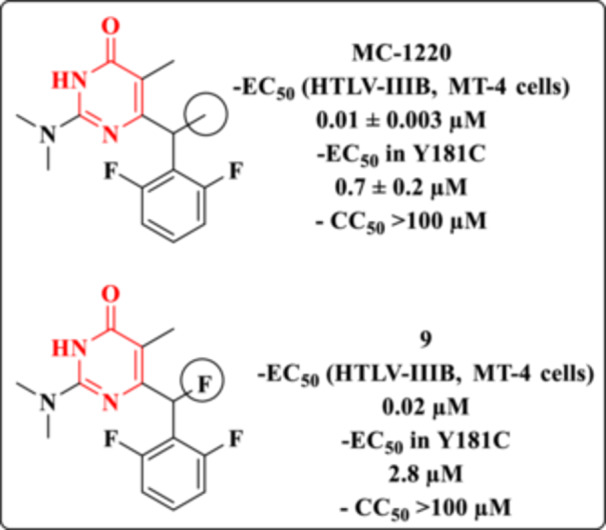
Chemical structure of the NNRTI, MC‐1220, and its Analog **9**.

Loksha et al. focused on the development of new analogs of MC‐1220 (Figure 15), a potent NNRTI activity [78]. MC‐1220, in its racemic form, displayed high efficacy against wild‐type HIV‐1 (EC_50_ = 0.01 ± 0.003 µM) and retained moderate activity against the clinically relevant Y181C mutant strain (EC_50_ = 0.7 ± 0.2 µM). Structural modifications were introduced to improve or retain potency while potentially enhancing pharmacological properties.

Among the synthesized analogs, Compound **9** emerged as the most promising derivative (Figure 15). This compound features the substitution of the methyl group in MC‐1220 with a fluorine atom. Despite this seemingly conservative modification, Compound **9** exhibited a slight reduction in antiretroviral activity, with EC_50_ values of 0.02 µM against the wild‐type HIV‐1 strain and 2.8 µM against the Y181C mutant. These results highlight the sensitivity of NNRTI activity and emphasize the importance of steric and electronic factors in modulating binding affinity to the RT enzyme [78].

With respect to the SI, Compound **9** exhibited a value greater than 5000, indicating a wide margin between antiviral activity and cytotoxicity. Although this result is highly favorable and consistent with the observed potency profile, the reference compound MC‐1220 displayed an even higher SI (> 10000), reflecting a superior balance between antiviral efficacy and cellular safety. This comparison suggests that, despite the slight reduction in antiretroviral potency observed for Compound **9** relative to the parent compound, the high SI confirms the retention of a promising pharmacological profile. Nevertheless, these data indicate that further structural optimization may be explored to achieve selectivity levels comparable to or exceeding those of the reference compound.

Conformationally restricted DHPM compounds were initially reported by Mai et al. in 2001 [79], following computational modeling that suggested enhanced HIV‐1 RT inhibitory activity and selectivity upon methylation of the benzylic (α) carbon at the C‐6 position of the 2,6‐difluorobenzylthymine scaffold [60]. This structural modification aimed to reduce conformational flexibility, thereby improving molecular recognition at the RT binding site. Motivated by these findings, the group subsequently investigated the introduction of a methoxy group at the same α‐position, acting as a more electron‐donating bioisostere of the methyl group. This substitution was anticipated to improve aqueous solubility without significantly increasing steric hindrance.

**Figure 16:**
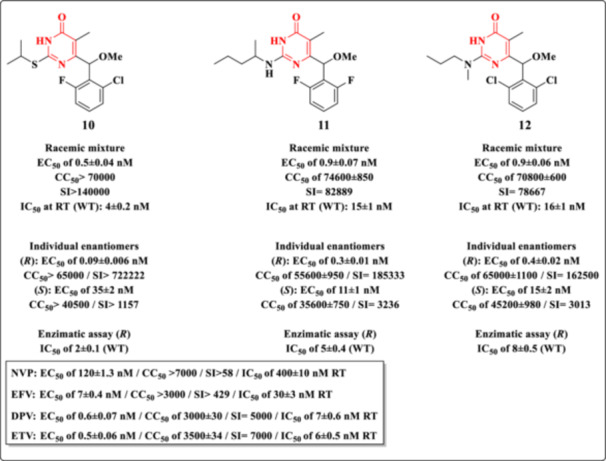
Antiretroviral profile of **10–12** DHPM compounds.

The synthesized α‐methoxy‐DHPM derivatives were evaluated for cytotoxicity and for their ability to inhibit the HIV‐1‐induced cytopathic effect in MT‐4 lymphoid cells (EC_50_; HIV‐1 strain NL4‐3). Among the tested analogs, Compounds **10–12** (Figure 16) emerged as the most potent, exhibiting inhibitory activity an order of magnitude greater than EFV and comparable to the reference NNRTIs dapivirine (DPV) and ETR, while demonstrating significantly reduced cytotoxicity toward MT‐4 cells.

For these three lead compounds, enantioseparation, chiroptical characterization, and assignment of absolute configuration were conducted. Evaluation of the individual enantiomers in both cellular and enzymatic assays revealed a pronounced enantioselectivity. The (*R*)‐enantiomers (eutomers) exhibited potent anti‐HIV‐1 activity at the picomolar level against wild‐type HIV‐1 and retained high inhibitory efficacy (in the nanomolar to submicromolar range) against key resistant RT variants, including K103N, Y181I, and L100I. In contrast, the (*S*)‐enantiomers (distomers) and the corresponding racemates displayed reduced potency. These findings underscore the critical role of stereochemistry at the C6‐benzylic position in modulating the antiviral activity of DHPM.

The evaluation of the SI revealed exceptionally high values for the investigated compounds, both in their racemic forms and after enantiomeric separation. The racemic mixture of Compound **10** exhibited an SI > 140000, whereas Compounds **11** and **12** showed SI values of 82,889 and 78,667, respectively. Notably, enantiomeric resolution led to a marked enhancement in selectivity, with the *R* enantiomer identified as the most active. In this context, Compound **10** ***R*** displayed an SI > 722,222, followed by Compounds **11** ***R*** (SI = 185,333) and **12** ***R*** (SI = 162,500). These values greatly exceed those observed for the reference drugs evaluated under similar conditions, including NVP (SI > 58), EFV (SI > 429), DPV (SI = 5000), and ETR (SI = 7000). Taken together, these findings highlight not only the critical role of stereochemistry in modulating antiviral selectivity but also the outstanding therapeutic potential of these compounds, which exhibit safety margins substantially superior to those of clinically used NNRTIs.

**Figure 17:**
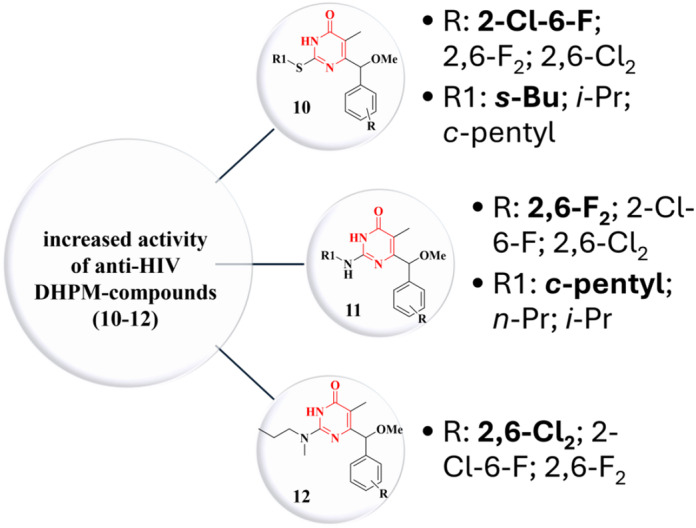
SAR of Derivatives **10–12**.

Molecular docking analysis of the eutomers' bound conformations revealed that the α‐methoxy substituent can establish additional van der Waals interactions within the NNBS of HIV‐1 RT. These interactions are proposed to contribute, at least in part, to the enhanced antiviral potency of the α‐methoxy DHPM derivatives relative to their α‐methyl counterparts. Nonetheless, this enhancement is not uniformly observed across all methoxy/methyl analog pairs, indicating that the impact of the methoxy group on activity is context‐dependent and influenced by the overall molecular architecture. A SAR summary for Derivatives **10–12** is presented in Figure 17.

**Figure 18:**
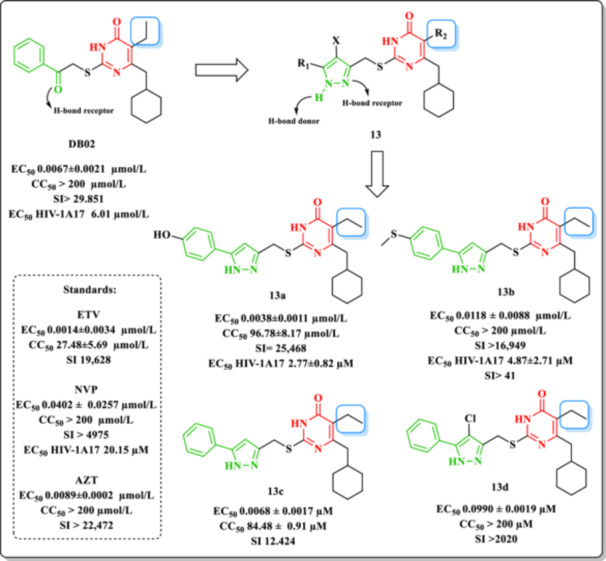
Design and inhibitory profile of HIV‐1 of DHPM Derivatives **13a–d** containing the pyrazole core.

Wu et al. previously reported a series of DHPM–molecules displaying potent anti‐HIV activity, among which compound DB02 was the most active, demonstrating efficacy against clinically relevant HIV‐1 RT‐resistant strains such as K103N and Y181C (Figure 18). The carbonyl moiety at the C‐2‐linked side chain was shown to form a key hydrogen bond at the RT binding site. To optimize this interaction, the authors proposed replacing the carbonyl with a pyrazole group, capable of acting as both a hydrogen bond donor and acceptor, potentially enhancing the binding affinity through reinforced ligand–receptor interactions [50, 56].

Based on this rationale, a series of DHPM–pyrazole derivatives were synthesized, incorporating various substituents of differing steric and electronic properties at the pyrazole moiety tethered to the C‐2 position of the DHPM scaffold. A total of 40 compounds were evaluated for antiviral efficacy and cytotoxicity in HIV_IIIB_‐infected C8166 cells, using ETR, NVP, and AZT as reference controls [50].

SAR analyses indicated that the substituent at the C‐2 side chain was the primary determinant of antiviral potency. The presence of a phenyl ring at R1 provided superior activity, likely due to favorable interactions at the enzyme–solvent interface, where longer and more hydrophobic groups are preferred. Molecular docking simulations further emphasized the importance of substitution at C‐5, as this modulated the orientation of the molecule within the RT hydrophobic pocket, directly impacting a crucial hydrogen bond between the pyrazole ring and the Lys103 residue. Notably, the presence of a methyl group at C‐5 facilitated optimal positioning of the molecule for this interaction.

Among the tested derivatives, Compounds **13a** (EC_50_ = 0.0038 ± 0.0011 µM; CC_50_ = 96.78 ± 8.17 µM) and **13b** (EC_50_ = 0.0118 ± 0.0088 µM; CC_50_ > 200 µM) exhibited the most favorable balance between antiviral activity and cellular safety (Figure 18). Consistent with these findings, Compound **13a** displayed an SI of 25,468, while Compound **13b** exhibited an SI greater than 16,949, indicating wide therapeutic windows and reinforcing their potential as promising NNRTI candidates with an advantageous efficacy‐toxicity profile.

Regarding the effect of halogenation at position X, derivatives bearing Cl or H were compared. Although overall activity differences were modest, certain substitutions had pronounced effects: for instance, Compound **13c** (X = H) displayed markedly greater potency (EC_50_ = 0.0068 ± 0.0017 µM) compared to its chlorinated Analog **13d** (X = Cl, EC_50_ = 0.0990 ± 0.0019 µM). Interestingly, Cl substitution was generally associated with reduced cytotoxicity: **13c** (CC_50_ = 84.48 ± 0.91 µM) versus **13d** (CC_50_ > 200 µM).

Based on these results, Compounds **13a** and **13b** were selected for further evaluation against resistant HIV‐1 strains. Against the multidrug‐resistant HIV‐1A17 strain carrying the K103N and Y181C mutations, both compounds retained inhibitory activity (EC_50_ = 2.77 and 4.87 µM, respectively), markedly outperforming NVP (EC_50_ = 20.15 µM). In terms of selectivity, Compound **13a** displayed an SI of 34, while Compound **13b** exhibited an SI > 41, values comparable to or higher than that of the reference compound **DB02** (SI > 33) and substantially superior to NVP (SI > 10), indicating a favorable therapeutic window.

In addition, enzymatic assays using recombinant HIV‐1 RT showed that Compound **13a** exhibited anti‐RT activity comparable to NVP (IC_50_ = 1.09 ± 0.59 mmol/L), although approximately threefold lower than **DB02** (IC_50_ = 0.28 ± 0.03 mmol/L), whereas Compound **13b** displayed weaker inhibition (IC_50_ = 2.27 ± 0.12 mmol/L). Both compounds demonstrated dose‐dependent inhibition profiles similar to those of NVP and **DB02**.

**Figure 19:**
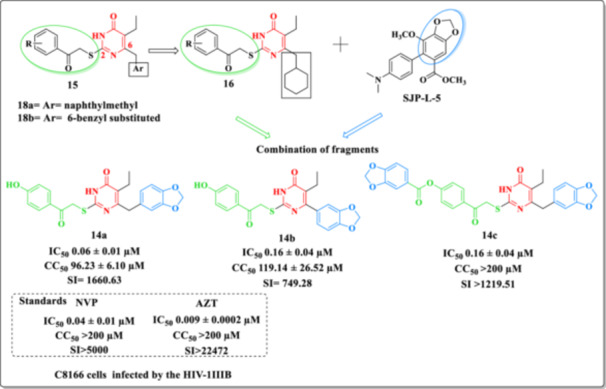
Design of Compounds **15** and **16** and chemical structures of analogs (**14a–c**) highlighting their anti‐HIV activity.

In the same year, Li et al. focused on the development of new analogs DHPM [62] based on previously performed structural optimizations, leading to the discovery of Compounds **14a–c** (Figure 19) with significant anti‐HIV‐1 activity [55, 71, 80, 81]. It is important to highlight that these compounds are based on the C‐2 and C‐6 positions of the DHPM ring, as these are the key sites responsible for modulating activity [49, 55, 57, 60, 72, 75].

In this context, the antiviral activity of different substituents at the C‐6 carbon of the DHPM ring in the new molecules was also evaluated. For instance, analogs bearing 6‐naphthylmethyl (**15a**), 6‐benzyl (**15b**), and 6‐cyclohexylmethyl (**16**) substituents showed satisfactory activity (Figure 19). Furthermore, molecular docking results indicated that the aromatic ring at the C‐6 carbon of Compounds **15a** and **15b** established favorable interactions with the side chains of Tyr181 and Tyr188 residues of RT, triggering positive π‐stacking interactions. Meanwhile, the cyclohexyl ring at the same position in Compound **16** was positioned within the upper hydrophobic cavity formed by Tyr181, Tyr188, Phe227, and Trp229 residues, favoring hydrophobic contacts with these residues.

In this study, the authors aimed to introduce a semi‐flexible group with a larger volume than cyclohexyl at the C‐6 position, anticipating that it would achieve a better fit within the hydrophobic pocket of the HIV‐RT enzyme. Therefore, the 1,3‐benzodioxole group was introduced at the C‐6 position of DHPM, considering that this group is an important component of SJP‐L‐5 (Figure 19), a compound derived from modifications of the dibenzocyclooctadiene lignan with antiretroviral activity.

Preliminary SAR analyses, supported by molecular docking studies, suggested that although these molecules can occupy the NNIBP, limited key interactions with critical aromatic residues may restrict their antiviral potency. These observations highlight the influence of subtle structural features on binding mode and mechanism of action, providing a rationale for further structural optimization of this compound series.

The most active analog, **14a**, exhibited HIV‐1 inhibitory activity with an IC_50_ value of 0.06 ± 0.001 µM, comparable to NVP (IC_50_ = 0.04 µM) but inferior to AZT (IC_50_ = 0.009 µM). Additionally, the compounds showed low cytotoxicity (Figure 19) [62].

The SI analysis revealed favorable values for the evaluated compounds, with **14a** exhibiting an SI of 1660.63, **14b** an SI of 749.28, and **14c** an SI greater than 1219.51, indicating a reasonable therapeutic window. Although these values are lower than those observed for the reference drugs NVP (SI > 5000) and AZT (SI > 22472), they nonetheless reflect an improved balance between antiviral activity and cytotoxicity compared to less selective candidates. Among the series, Compound **14a** showed the most advantageous selectivity profile, supporting its further optimization as a potential antiviral lead.

Given the promising results obtained in the wild‐type cell line, the authors proceeded to evaluate the in vitro activity of Compounds **14a–c** against various resistant HIV‐1 strains, including protease inhibitor‐resistant strains (HIV‐1RF/V82F/184V), fusion inhibitor‐resistant strain (pNL4‐3gp41(36G)V38A,N42T), NRTI‐resistant strain (HIV‐14755‐5), NNRTI‐resistant strain (HIV‐1A17), and single point mutations in HIV‐1 RT (HIV‐1K103N and HIV‐1Y181C), as well as against clinical isolates (HIV‐1TC‐1, HIV‐1WAN, HIV‐1KIZ001, and HIV‐1KM018) [62]. The results demonstrated that all three analogs exhibited potent inhibitory activity against protease inhibitor‐, fusion inhibitor‐, and NRTI‐resistant HIV‐1 strains. However, their activity was reduced against NNRTI‐resistant strains. Furthermore, Compounds **14a–c** showed significant inhibition of replication in clinical HIV‐1 isolates and exhibited low cytotoxicity in peripheral blood mononuclear cells (PBMCs).

Compounds **14a** and **14b** were found to inhibit both RNA‐dependent (IC_50_ of **14a** = 0.518 ± 0.057 µM; IC_50_ of **14b** = 1.532 ± 0.675 µM) and DNA‐dependent DNA polymerase activities of HIV‐1 RT (IC_50_ of **14a** = 0.072 ± 0.020 µM; IC_50_ of **14b** = 0.126 ± 0.023 µM), resembling the mechanism of classical RT inhibitors. In contrast, Compound **14c** selectively inhibited DNA‐dependent DNA polymerase activity (IC_50_ = 2.180 ± 8.181 µM), despite its structural similarity to Compounds **14a** and **14b**.

**Figure 20:**
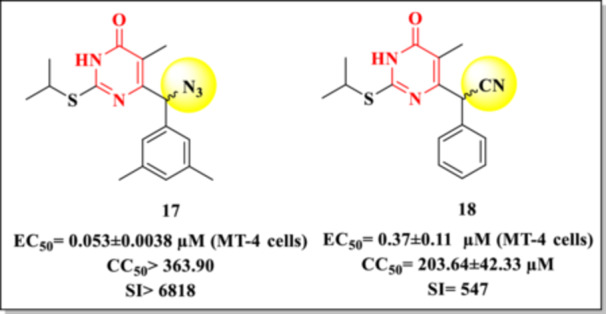
The chemical structure of DHPM compounds, **17** and **18**.

Recently, Ling et al. [82] developed a highly promising DHPM molecule (**17**) by substituting the cyano group of the previously reported Compound **18** [83] with an azido group—a moiety also present in the structures of two well‐known NRTIs, AZT and FNC. Molecular docking simulations indicated that the azido‐modified derivative maintained a conformation like its Prototype **18** within the allosteric binding pocket of the HIV‐1 RT, with the primary difference being the orientation of the N₃ group relative to the CN group in Compound **18**. This structural modification led to a sevenfold increase in anti‐HIV activity and a 12.5‐fold improvement in selectivity against wild‐type HIV‐1 strains (Figure 20).

In enzymatic assays, the azido‐substituted compound also exhibited superior inhibitory potency against wild‐type RT, with an IC_50_ value of 0.080 ± 0.010 µM, compared to 1.51 ± 0.11 µM for Compound **18**. Furthermore, in vivo assays demonstrated low inhibitory activity against hERG channels and cytochrome P450 enzymes (CYP), supporting a favorable safety and pharmacological profile for further development.

In 2015, Sari et al. synthesized a series of DHPM derivatives based on α,β‐diketo butanoic acid scaffolds, aiming to inhibit HIV IN Strand Transfer (ST) activity [84]. The study encompassed two series of novel compounds, totaling 20 prototypes, whose enzymatic inhibitory activities were evaluated and compared to the reference drug RAL. SAR analysis revealed that substitution at the C‐6 position of the DHPM core plays a critical role in determining antiretroviral potency. Bulky substituents at this position, such as phenyl or isopropyl groups, resulted in reduced activity, likely due to steric hindrance. Conversely, smaller groups like methyl or ethyl enhanced the inhibitory effect on the enzyme.

**Figure 21:**
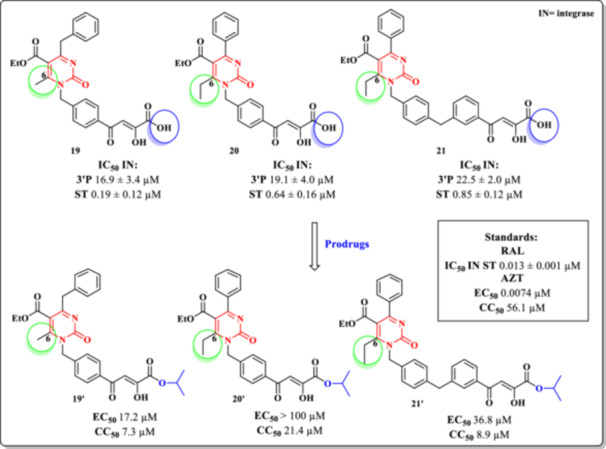
Chemical structures and anti‐HIV‐1 profile of DHPM Derivatives **19–21** and their respective prodrugs **19′–21′**.

Among the synthesized compounds, the most promising IIs were Compounds **19**, **20**, and **21**, which exhibited submicromolar IC₅₀ values of 0.19 ± 0.12, 0.64 ± 0.16, and 0.85 ± 0.12 µM, respectively (Figure 21). Despite their potent enzymatic activity, these compounds failed to demonstrate efficacy in cell‐based assays using CEM cells, with EC_50_ values exceeding 65 µM, suggesting poor cellular uptake. The authors attributed this discrepancy to the high polarity of the carboxylic acid group, which limited membrane permeability.

To overcome this limitation, the most active compounds were converted into ester prodrugs (designated **19′–21′**), designed to undergo enzymatic hydrolysis by hepatic esterases, thereby releasing the active acid form intracellularly. Following metabolism, the generated active compounds demonstrated antiviral activity in CEM cells. However, some of the prodrugs exhibited undesirable cytotoxicity, raising concerns regarding their therapeutic window. The authors do not report or discuss the values of the corresponding SI. AZT was used as the reference standard in these cellular assays (Figure 21).

In the same year, Naidu et al. reported the synthesis of 35 DHPM–carboxamide derivatives designed to inhibit the ST step of the HIV‐1 IN catalytic cycle [14]. The compounds were evaluated for anti‐HIV activity in cellular assays using fetal bovine serum (FBS), and their pharmacokinetic profiles and binding affinities to human serum albumin (HSA) were also assessed.

The structural design featured carboxamide moieties at the C‐4 position of the DHPM core and various heterocyclic substituents at C‐2, notably tetrahydrofuran (THF) and tetrahydropyran (THP) rings. These modifications yielded promising antiviral profiles. Among the THF‐containing derivatives, Compound **22a** exhibited the most potent activity, with an IC_50_ of 0.004 µM against IN and an EC_50_ of 0.009 µM in cellular assays. Similarly, in the THP series, Compound **22b** stood out, displaying an IC_50_ of 0.005 µM and an EC_50_ of 0.006 µM. Both compounds demonstrated enhanced hydrophilicity, which contributed to reduced binding to plasma proteins, notably HSA.

**Figure 22:**
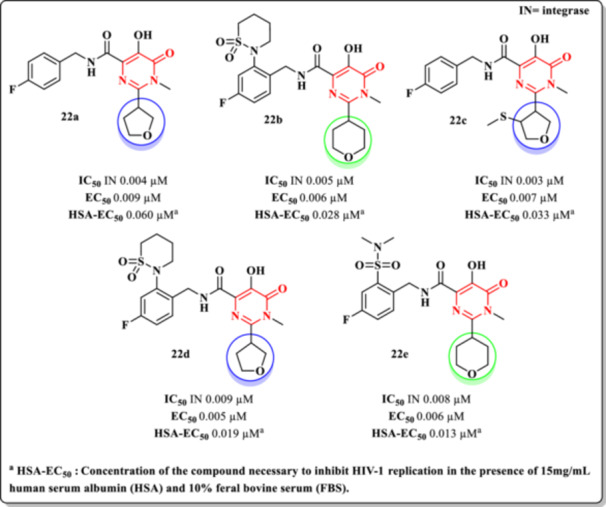
Chemical structure of DHPM–carboxamide Derivatives **22a–e** and their anti‐HIV activities.

The authors proposed that the oxygen atom within the THP ring may participate in additional hydrogen bonding interactions at the IN binding site, thereby improving receptor affinity and overall inhibitory activity (Figure 22). Further SAR exploration revealed that specific substitutions on the THF ring could further enhance potency. Compound **22c**, bearing such modifications, exhibited superior enzymatic inhibition (IC_50_ = 0.003 µM; EC_50_ = 0.007 µM), outperforming Compound **22a** in both enzymatic and cellular assays. Additionally, it showed a lower degree of albumin binding.

Despite its high enzymatic potency and favorable pharmacokinetic parameters, Compound **22a** suffered a notable reduction in activity when tested in the presence of albumin, highlighting the impact of protein binding on bioavailability. To address this issue, the authors introduced modifications at the ortho position of the benzylamine moiety to reduce HSA affinity. Among these, Compound **22d** exhibited potent antiviral activity (IC_50_ = 0.009 µM; EC_50_ = 0.005 µM) along with favorable pharmacokinetic attributes, including high oral bioavailability. Conversely, Compound **22e** demonstrated strong in vitro activity (IC_50_ = 0.008 µM; EC_50_ = 0.006 µM) but still suffered reduced efficacy due to high albumin binding (Figure 22). The authors do not report or discuss the values of the corresponding SI.

In a subsequent study, Naidu et al. further investigated the DHPM–carboxamide scaffold by introducing diverse substituents at the C‐2 position of the heterocycle [85]. Among the evaluated modifications, the incorporation of a morpholine ring at this position led to a compound with markedly improved aqueous solubility compared to other heterocyclic analogs. Additionally, the morpholine moiety contributed to reduced plasma protein binding and minimized potential for cytochrome P450 (CYP450) inhibition—key parameters for optimizing pharmacokinetic behavior.

**Figure 23:**
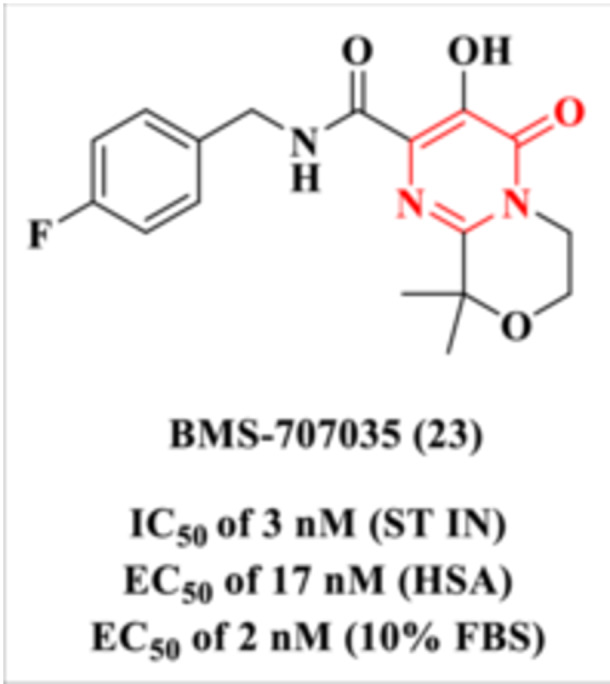
Chemical structure of the integrase ST inhibitor (INSTI) developed by Naidu et al. [85].

These combined features culminated in the identification of BMS‐707035 (Compound **23**), which emerged as a lead candidate (Figure 23). This compound demonstrated potent inhibition of the IN ST step and retained significant antiviral activity even under conditions that simulate physiological protein binding, including assays conducted in the presence of 15 mg/mL HSA or 10% FBS, as shown in Figure 23.

Compound **23**, a potent HIV‐1 INSTI, exhibited excellent antiviral activity with an EC_50_ of 3 nM and demonstrated an acceptable preclinical toxicity profile in both in vitro and in vivo models. These favorable characteristics supported its progression to Phase I clinical trials in healthy human volunteers [85]. The compound was administered as single oral doses of 200, 600, 800, and 1200 mg, all of which were well tolerated, with no clinically significant adverse events reported. Among these, the 600 mg dose was established as the effective pharmacological dose, capable of achieving plasma concentrations sufficient to significantly inhibit HIV‐1 replication.

Despite its promising clinical potential, the development of Compound **23** was ultimately discontinued. This decision was based on findings from a 12‐month toxicology study, in which two dogs experienced seizures at high peak plasma exposures (*C*_max_ ≥ 67 μg/mL). These adverse neurological events raised safety concerns that precluded further clinical advancement of the compound.

**Figure 24:**
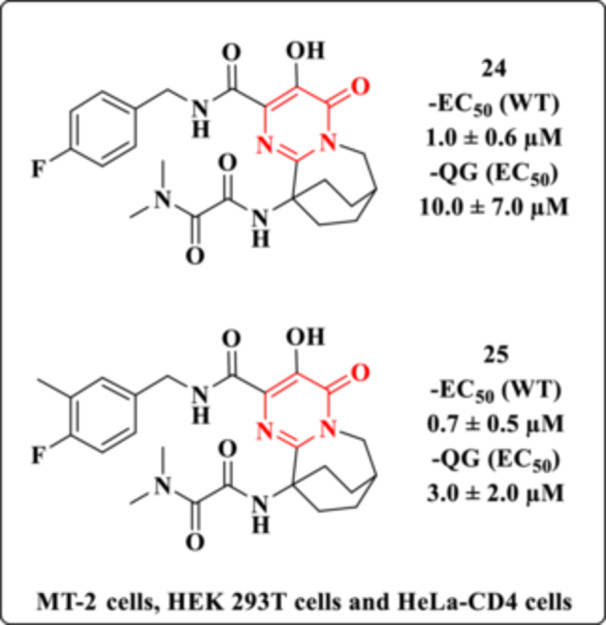
Bridged tricyclic pyrimidinone carboxamides as HIV‐1 integrase inhibitors.

Subsequently, following an extensive screening of tricyclic analogs, Patel et al. identified Compounds **24** and **25** as promising chemotypes displaying notable antiviral activity against both wild‐type viruses and the G140S/Q148H (GQ) resistant mutant strain (Figure 24) [86]. SAR studies were conducted to elucidate the role of the oxamide moiety in mediating inhibitory activity against the GQ mutant, with the following key findings:

- The removal of one carbonyl group from the oxamide moiety in Compound **24** caused a marked reduction in antiviral activity against the GQ mutant, while maintaining considerable potency against the wild‐type virus. Likewise, replacing the *N*,*N*‐dimethylamide with an ester led to diminished inhibitory activity against GQ.
- Substitution of the nitrogen atom in the *N*,*N*‐dimethylamide portion with a methylene group retained moderate inhibitory activity against GQ, whereas insertion of a methylene spacer between the oxamide carbonyls significantly impaired potency against the resistant virus.
- Modifications to the terminal nitrogen atom generally did not affect wild‐type virus inhibition but had a significant impact on activity against the GQ mutant.
- Removal of one methyl substituent from the *N*,*N*‐dimethyloxamide group resulted in a 65‐fold decrease in potency against GQ. Conversely, replacing one or both methyl groups with ethyl, trifluoroethyl, isopropyl, cyclopropyl, or isobutyl groups was well tolerated.
- Analysis of substituents on the benzylamide fragment, while retaining the *N*,*N*‐dimethylacetamide at the bridgehead position, revealed that small meta‐substituents such as fluorine, chlorine, and methyl enhanced inhibitory potency against GQ by two to eightfold.
- Conversely, increased steric bulk at the ortho position correlated with reduced inhibitory potency against GQ. This decrease is attributed, in part, to unfavorable conformational changes caused by steric clashes between the ortho substituent and the *N*,*N*‐dimethylacetamide, which are proposed to compete for the same pharmacophoric site on the IN protein.

In vitro ADME studies of Compound **25** analogs further underscored the critical role of the oxamide moiety in maintaining inhibitory activity against the GQ mutant strain [86]. However, pharmacokinetic evaluation of Compound **25** revealed an unfavorable profile for human dosing, compounded by glucuronidation as the predominant metabolic pathway. Consequently, the clinical development of this compound was discontinued due to low prospects for therapeutic success [86].

**Figure 25:**
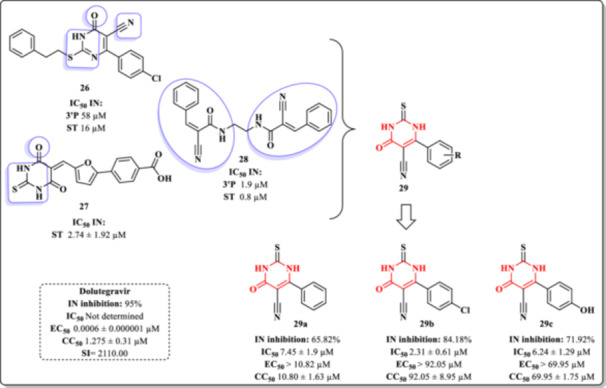
Design of DHPM Derivatives **29a–c** and their inhibitory activity profile against HIV‐1_IIIB_ and cytotoxicity in MT‐4 cells.

Wadhwa et al. developed a series of derivatives featuring a DHPM core (Figure 25) [87]. The design strategy was based on previously reported compounds exhibiting significant inhibitory activities against the HIV‐1 IN enzyme: Compound **26**, which demonstrated inhibition of both IN catalytic steps—3′‐processing (IC_50_ = 58 µM) and ST (IC_50_ = 16 µM) [88]; Compound **27**, which showed potent inhibition of ST with an IC_50_ of 2.74 µM [89]; and Compound **28**, a tyrosine kinase inhibitor known to inhibit both IN activities, with ST and 3′‐processing IC_50_ values of 0.8 and 1.9 µM, respectively [90]. Using these compounds as lead structures, the Wadhwa group synthesized Derivatives **29a–c** (Figure 25) [87].

The inhibitory activity of Compounds **29a–c** against the IN enzyme was assessed in vitro using the Xpress Bio kit protocol (Xpressbio Life Science Products, USA), with dolutegravir (DTG) serving as the positive control. Additionally, anti‐HIV activity and cytotoxicity were evaluated in MT‐4 cells infected with the HIV‐1_IIIB_ strain.

Out of 24 synthesized derivatives, IC_50_ values were determined only for those exhibiting promising inhibitory activity in the assays. Compounds possessing either an unsubstituted phenyl group (**29a**) or para‐substituted phenyl groups (**29b** and **29c**) showed notable inhibition (Figure 25). Specifically, Compound **29a** achieved 65.82% inhibiting of IN activity with an IC_50_ of 7.45 µM; Compound **29b** showed 84.18% inhibition with an IC_50_ of 2.3 µM; and Compound **29c** exhibited 71.02% inhibition with an IC_50_ of 6.24 µM. Despite these results, none of the compounds demonstrated significant antiretroviral activity in cellular assays at concentrations below their respective cytotoxic thresholds (Figure 25). This behavior revealed unfavorable SI profiles for compounds, all of which exhibited SI values below 1.

Another critical step for HIV entry into host cells involves the interaction between the viral envelope glycoprotein gp120 and the cellular CD4^+^ receptor. A primary target for disrupting this interaction is the hydrophobic pocket of gp120—known as the Phe43 cavity—which accommodates the phenyl group of the CD4^+^ residue Phe43 [19]. To block this interaction, Senapathi et al. designed small molecules based on the DHPM scaffold [91].

The authors drew inspiration from natural alkaloids previously identified as ligands for the gp120‐CD4^+^ binding site. Structural modeling studies suggested that the DHPM scaffold could facilitate hydrogen bonding between the amide of the guanidine ring and the carboxylic acid group of Asp368, a residue lining the Phe43 cavity.

**Figure 26:**
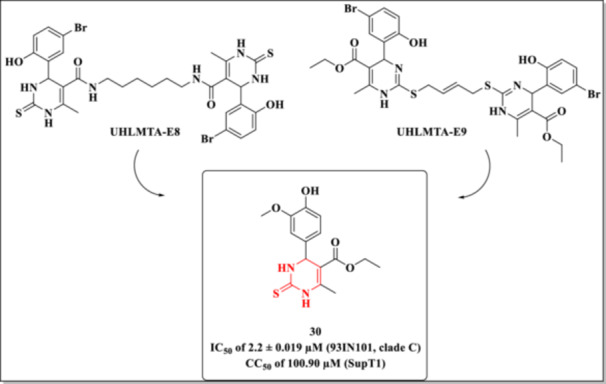
HIV entry inhibition potential of compounds synthesized by Senapathi et al. [91].

Before developing new DHPM analogs, molecular docking simulations were conducted using prototype compounds UHLMTA‐E8 and its derivative UHLMTA‐E9 (Figure 26) docked to the gp120 protein. These simulations revealed that neither compound exhibited favorable interactions within the Phe43 cavity. Consequently, the authors proposed structural modifications aimed at enhancing the potential to form a salt bridge with Asp368, optimizing antiviral activity by restricting molecular flexibility and length [91].

Two series of compounds were synthesized: DHPMs and DHPM–thiones. The latter, which incorporates a sulfur atom in the pyrimidine ring, demonstrated superior antiviral activity relative to their oxygen‐containing counterparts, albeit with increased cytotoxicity. Among them, Compound **30** (Figure 26) emerged as the most potent. However, the authors do not report or discuss the values of the corresponding SI. Molecular docking simulations confirmed the formation of a key hydrogen bond between Compound **30** and Asp368 in gp120—a critical interaction required for CD4^+^ recognition and subsequent membrane fusion during viral entry.

**Figure 27:**
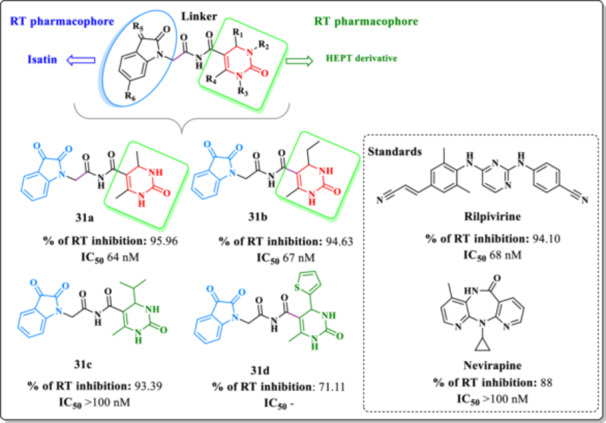
Design of the new Analogs **31a–d** and their RT inhibitory profile.

Devale et al. designed a series of hybrid compounds (**31a–d**) by combining two pharmacologically privileged scaffolds active at RT (Figure 27) [92]. The objective was to develop novel molecules with inhibitory activity against HIV‐1 RT. The authors successfully synthesized a series of DHPM–isatin hybrids, which were subsequently evaluated for their RT inhibitory potential. Among these, Compounds **31a** and **31b** demonstrated superior inhibitory activity compared to the reference NNRTIs RPV and NVP, indicating their promise as lead compounds.

SAR analysis revealed that small aliphatic substituents at the R^1^ position, such as those found in Compounds **31a–c**, were more favorable for RT inhibition than bulkier aromatic or heteroaromatic groups, as exemplified by Compound **31d**. Furthermore, the isatin moiety appeared to be critical for maintaining activity, and its modification led to reduced potency. These findings suggest that careful preservation of the isatin core, along with optimization of small aliphatic groups at specific positions, may enhance the anti‐RT potential of these hybrid molecules.

**Figure 28:**
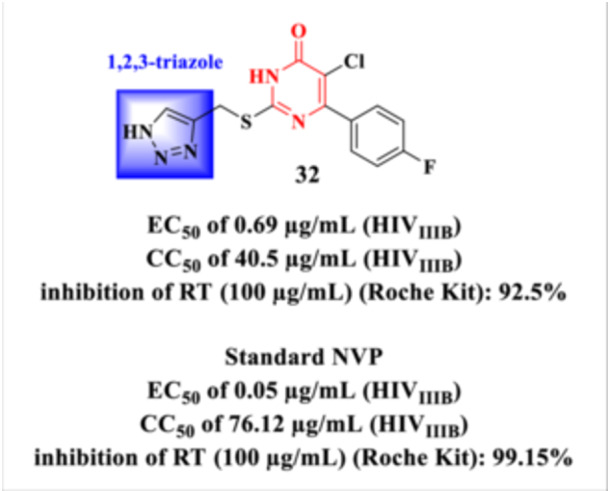
Antiretroviral profile of the Hybrid **32**.

Kasralikar et al. employed a molecular hybridization strategy by combining two pharmacologically relevant heterocyclic scaffolds [93]: 1,2,3‐triazole and DHPM, supported by extensive literature describing the broad pharmacological applicability of these classes (Figure 28) [94, 95, 96]. The rationale behind this approach was to develop novel hybrid molecules with enhanced selectivity and potency against HIV‐1 RT.

The study highlighted that selective inhibition of RT could be achieved through key structural interactions. Specifically, the hydrogen atom at the *N*‐1 position of the 1,2,3‐triazolyl moiety in Compound **32** was shown to form a hydrogen bond with the backbone N–H group of the Lys101 residue in the RT active site. Loss of this interaction correlated with a marked reduction in anti‐HIV activity. Additionally, the fluorine‐substituted aromatic ring present in the thiouracil moiety could engage in π–π stacking interactions within the hydrophobic pocket defined by the aromatic side chain of Trp229. Furthermore, the incorporation of a chlorine atom on the thiouracil ring increased the overall hydrophobicity of the compound, contributing to improved anti‐HIV activity. These findings underscore the importance of hydrogen bonding, π–π stacking, and hydrophobic interactions in the rational design of RT inhibitors based on hybrid triazole–DHPM scaffolds.

The biological results indicate that the tested Compound **32** exhibits relevant antiviral activity, with an EC_50_ value of 0.69 µg/mL associated with moderate cytotoxicity (CC_50_ = 40.5 µg/mL), resulting in a favorable therapeutic margin (SI = 58.69). In addition, the compound promoted a marked inhibition of RT, reaching 92.5% inhibition at a concentration of 100 µg/mL, thereby reinforcing its potential as a promising candidate for further optimization and mechanistic studies.

**Figure 29:**
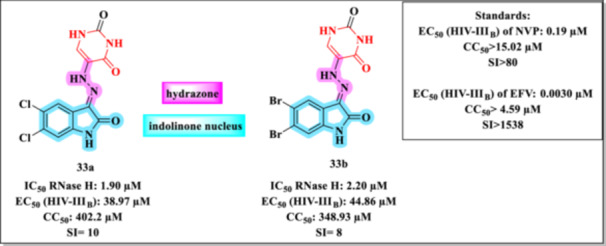
3‐Hydrazonoindolin‐2‐one Derivatives **33a–b**.

Zhang et al. recently reported a novel class of 3‐hydrazonoindolin‐2‐one derivatives designed to inhibit the RNase H activity of HIV‐1 RT [97]. The study involved the synthesis of a series of new compounds based on the indolinone scaffold, bearing hydrazone substitutions at position 3 (Figure 29). Notably, Compounds **33a** and **33b** demonstrated significant in vitro RNase H inhibitory activity, with IC_50_ values of 1.90 and 2.20 µM, respectively.

In addition to enzymatic assays, the compounds were also evaluated for their anti‐HIV activity against the HIV‐1 strain IIIB in MT‐4 cells, alongside cytotoxicity assessments (Figure 29). The biological results indicate that Compounds **33a** and **33b** exhibited moderate antiviral activity, with EC_50_ values of 38.97 and 44.86 µM, respectively, accompanied by low cytotoxicity, as reflected by CC_50_ values of 402.2 µM for **33a** and 348.93 µM for **33b**. Consequently, both compounds displayed modest SI (10 for **33a** and 8 for **33b**), suggesting a limited therapeutic window, although consistent with early‐stage lead profiles.

The molecular design incorporates hydrophilic groups ‐ introduced via the hydrazone moiety—for interaction with the catalytic metal ions, as well as lipophilic regions that facilitate binding within the enzyme's hydrophobic pocket.

Molecular modeling studies suggest that the hydrazone moiety contributes to the stabilization of coordination with Mg^2+^ ions at the RNase H active site. SAR analysis revealed that modifications in the hydrazone side chain markedly affect enzymatic affinity, underscoring the importance of structural optimization for enhancing inhibitory potency.

As demonstrated throughout the text, the DHPM core has emerged as a valuable scaffold in the search for novel HIV inhibitors, owing to its structural versatility and capacity to interact with key molecular targets in the viral life cycle, particularly RT. DHPM‐based compounds—especially those bearing strategically positioned substituents—have shown notable affinity for the hydrophobic regions of the enzyme's allosteric site, acting as NNRTIs. This class of molecules offers the possibility of fine‐tuning physicochemical and pharmacokinetic properties, which is essential for the development of more potent and selective drug candidates with a lower risk of resistance development. Therefore, evaluating the drug‐like properties of representative DHPM derivatives will be crucial for the rational design of next‐generation anti‐HIV agents.

## 4 In Silico Prediction of Physicochemical Properties

To be considered a promising oral drug candidate, a molecule must possess a set of physicochemical properties that support its biological activity, chemical stability, absorption, distribution, and an acceptable toxicity profile [98]. The DHPM core can contribute to the improvement of pharmacokinetic parameters in orally administered drugs; however, its impact is not universal and depends on the structural and functional context of the compound. Nevertheless, several intrinsic features of the DHPM scaffold can positively influence key parameters:

- Enhanced solubility: The carbonyl group and the nitrogen atoms in the DHPM ring can engage in hydrogen bonding (as both donors and acceptors), potentially increasing aqueous solubility, which is essential for efficient oral absorption.
- Improved permeability: The relatively planar and partially aromatic structure of DHPM may favor passive diffusion across biological membranes, enhancing intestinal absorption.
- Chemical and metabolic stability: The pyrimidinone ring can offer resistance to metabolic degradation by hepatic enzymes, thereby contributing to prolonged plasma half‐life and improved oral bioavailability.
- Structural versatility: The availability of multiple substitution sites on the DHPM ring allows for the introduction of functional groups that modulate lipophilicity, polarity, and affinity for intestinal transporters, facilitating pharmacokinetic optimization.

To further investigate the drug‐like properties of the representative DHPM compounds, a preliminary evaluation of key physicochemical parameters was conducted using the online platform ADMETlab 3.0 [99]. As summarized in Table 1, all analyzed molecules met the criteria outlined in Lipinski's Rule of Five, including molecular weight (MW < 500 Da), number of hydrogen bond acceptors (nON < 10), number of hydrogen bond donors (nOHNH < 5), and partition coefficient (log *P* < 5). These parameters are widely recognized as indicators of optimal oral bioavailability. According to the rule, one violation is acceptable; however, compounds with two or more violations may exhibit poor absorption or permeability [99].

**Table 1:** Physicochemical properties of DHPM compounds described.

Compound	Parameters				
	MW	nON	nOHNH	Log *P*	tPSA
**3a**	429.07	5	1	4.39	78.77
**4**	316.12	4	2	2.37	65.98
**5**	504.04	4	1	4.63	62.82
**6**	532.14	9	3	2.15	136.62
**7**	550.04	9	3	2.45	136.62
**8**	452.21	5	1	6.17	72.05
**9**	297.11	4	1	2.29	48.99
**10**	356.08	4	1	3.59	54.98
**11**	351.18	5	2	3.60	67.01
**12**	369.10	5	1	3.97	58.22
**13a**	424.19	6	3	4.29	94.66
**13b**	454.19	5	2	5.24	74.43
**13c**	408.2	5	2	4.44	74.43
**13d**	442.16	5	2	4.81	74.43
**14a**	424.11	7	2	2.75	101.51
**14b**	410.09	7	2	3.16	101.51
**14c**	572.13	10	1	3.92	126.04
**17**	343.15	6	1	4.03	94.51
**18**	299.11	4	1	3.42	69.54
**19**	476.16	9	2	2.68	135.79
**20**	476.16	9	2	2.65	135.79
**21**	566.21	9	2	3.98	135.79
**22a**	347.13	7	2	1.46	93.45
**22b**	494.16	10	2	1.56	130.83
**22c**	393.12	7	2	1.87	93.45
**22d**	480.15	10	2	1.49	130.83
**22e**	468.15	10	2	1.40	130.83
**23**	347.13	7	2	1.78	93.45
**24**	471.19	10	3	1.32	133.63
**25**	485.21	10	3	1.94	133.63
**29a**	229.03	4	2	1.18	72.44
**29b**	262.99	4	2	1.89	72.44
**29c**	245.03	5	3	1.00	92.67
**30**	322.1	6	3	1.24	79.82
**31a**	356.11	9	3	0.50	124.68
**31b**	370.13	9	3	0.85	124.68
**31c**	384.14	9	3	0.81	124.68
**31d**	424.08	9	3	1.09	124.68
**32**	337.02	6	2	2.08	87.32
**33a**	338.99	8	4	1.76	119.21
**33b**	426.89	8	4	1.83	119.21
**ETV**	434.05	7	3	5.2^a^	120.64
**RPV**	366.16	6	2	4.86^a^	97.42

*Note:* Values highlighted in red indicate those that fall outside Lipinski's Rule of Five.

^a^ Values obtained from [100].

In addition, the topological polar surface area (tPSA) of all compounds fell within acceptable limits, supporting favorable intestinal absorption (tPSA < 140 Å^2^). Notably, most tPSA values were above 60 Å^2^, suggesting a low likelihood of blood–brain barrier (BBB) penetration, which reduces the risk of central nervous system‐related side effects.

To date, no marketed NNRTI incorporates a DHPM scaffold. However, two pyrimidine‐based derivatives—ETV and RPV—are well‐known members of the diarylpyrimidine (DAPY) class. Both compounds exhibit high log *P* values (log *P* to ETV: 5.2; log *P* to RPV: 4.86) [100], which are associated with prolonged half‐lives in vivo and enhanced cellular membrane permeability. Nevertheless, their low aqueous solubility imposes limitations on oral administration, necessitating specific formulation strategies to ensure effective absorption [100].

As shown in Table 1, except for Compounds **8** and **13b**, all other analogs demonstrate a potentially improved lipophilicity profile, as indicated by lower log *P* values compared to those of ETV and RPV. This characteristic may translate into enhanced solubility, representing a favorable pharmacokinetic trait in the context of drug development.

## 5 Conclusion

The DHPM core plays a pivotal role in the rational design of antiretroviral agents due to its ability to mimic nucleotide bases, enabling interactions with key viral enzymes such as HIV‐RT. This scaffold exhibits high structural versatility, allowing for targeted modifications that support the development of both NRTIs and NNRTIs. Beyond RT inhibition, DHPM‐based compounds have also demonstrated the capacity to interact with HIV IN, thereby disrupting the viral replication cycle without interfering with host cellular mechanisms.

The attractiveness of DHPMs in drug development stems from their potential for optimization in terms of potency, selectivity, and pharmacokinetic properties, including metabolic stability and oral bioavailability. Furthermore, their structural flexibility offers a strategic advantage in overcoming drug resistance, as specific substitutions within the DHPM scaffold can circumvent RT mutations that typically compromise the efficacy of conventional antiretrovirals. As such, the DHPM nucleus represents a valuable chemical platform for the development of innovative and effective HIV therapies.

In addition, in silico predictions of physicochemical parameters were performed to assess the drug‐like properties of the DHPM derivatives included in this review. All evaluated compounds demonstrated favorable characteristics, including high antiviral activity, low cytotoxicity, and compliance with key drug‐likeness criteria. These findings support the use of DHPM‐based molecules as promising leads for further structural refinement in the pursuit of potent HIV inhibitors.

## 6 Future Directions

Recent advances described for compounds containing the pyrimidinone core confirm this scaffold as a versatile and highly promising platform for the development of new antiretroviral agents, particularly NNRTIs. Nevertheless, the available data also highlight structural and pharmacokinetic challenges that should guide future investigations.

From a structural optimization perspective, future studies should further explore the rational modulation of the C‐2, C‐5, and C‐6 positions of the pyrimidinone nucleus, which have proven to be critical for affinity and selectivity toward the NNIBP. The systematic integration of molecular docking and MD data will allow correlations between electronic and steric modifications and ligand‐induced conformational changes, clarifying the role of key interactions such as halogen bonding with Tyr181, hydrogen bonding with Lys101 and Val106, and π–π interactions with conserved aromatic residues (Tyr181, Tyr188, Trp229, and Phe227). This approach is particularly relevant for understanding activity differences among closely related series, such as S‐DABOs and their C‐2 oxygenated analogs.

Another strategic direction involves the development of compounds active against resistant strains, as the inherent conformational flexibility of pyrimidinone derivatives can be exploited to accommodate mutations within the NNIBP. The introduction of substituents capable of maintaining alternative interaction networks may help mitigate the loss of activity commonly observed with classical RT mutants.

With respect to pharmacokinetic and safety profiles, future research should move beyond in vitro potency and address recurring limitations such as poor cellular permeability, high plasma protein binding, and potential off‐target effects. Strategies including prodrug design, fine‐tuning of lipophilicity, and incorporation of balanced polar functionalities may improve bioavailability without compromising target affinity. Early evaluation of ADMET parameters and cardiac safety (e.g., hERG liability) will be essential to enhance the translational potential of these compounds.

Additionally, the pyrimidinone scaffold offers opportunities for the design of multitarget molecules, combining RT inhibition with activity against other stages of the HIV life cycle, such as IN function or viral entry processes. Molecular hybridization and computational design strategies may yield compounds capable of interfering with multiple viral pathways, thereby reducing the risk of resistance development.

Finally, in the context of current antiretroviral therapy paradigms, pyrimidinone‐based derivatives should be evaluated for their suitability in long‐acting formulations and combination regimens. The integration of medicinal chemistry, advanced molecular modeling, and robust biological evaluation will be crucial to establish the pyrimidinone nucleus as a foundation for the next generation of more effective, selective, and clinically viable antiretroviral agents.

## Funding

The authors received no specific funding for this work.

## Ethics Statement

The authors have nothing to report.

## Consent

The authors have nothing to report.

## Conflicts of Interest

The authors declare no conflicts of interest.

## Data Availability

The authors have nothing to report.
